# Down‐regulation of miRNA‐27b‐3p suppresses keratinocytes apoptosis in oral lichen planus

**DOI:** 10.1111/jcmm.14324

**Published:** 2019-04-11

**Authors:** Junjun Chen, Yufeng Wang, Guanhuan Du, Wenyi Zhang, Tianyi Cao, Linjun Shi, Yanni Wang, Jun Mi, Guoyao Tang

**Affiliations:** ^1^ Department of Oral Medicine Shanghai Ninth People's Hospital, Shanghai Jiao Tong University School of Medicine Shanghai China; ^2^ Department of Biochemistry & Molecular Cell Biology, Shanghai Key Laboratory of Tumor Microenvironment and Inflammation Shanghai Jiao Tong University School of Medicine Shanghai China

**Keywords:** apoptosis, cyclophilin D, epithelium, miR‐27b‐3p, Oral Lichen Planus

## Abstract

Oral lichen planus (OLP) is considered a precancerous lesion with no known cure. Recent studies reported that abnormal regulation of apoptosis was involved in the pathogenesis of OLP. Next generation sequencing was used to screen the candidate microRNAs and genes in biopsies from patients with OLP and healthy mucosa. Human oral keratinocytes were transfected into the related oligonucleotides of miR‐27b‐3p/cyclophilin D and their control groups. Apoptosis was detected by TdT‐mediated dUTP nick end labelling and flow cytometry. The levels of mRNA and protein were detected by quantitative PCR, Western blots, and enzyme‐linked immunosorbent assays, respectively. Luciferase assays were performed to detect the luciferase activities of miR‐27b‐3p and cyclophilin D. Here, we showed that basal epithelium apoptosis was reduced and the miR‐27b‐3p levels were decreased in clinical OLP samples. We also found that down‐regulation of miR‐27b‐3p inhibited epithelial keratinocyte apoptosis by up‐regulating cyclophilin D expression. Moreover, cyclophilin D increased the protein stability of Bcl2 through direct binding, and Bcl2 suppressed caspase9/3 activation and cytochrome C release. Taken together, these data showed that miR‐27b‐3p regulated keratinocyte apoptosis through cyclophilin D/Bcl2 signalling, suggesting the miR‐27b‐3p regulated the pathogenesis of OLP.

## 1 INTRODUCTION

Oral lichen planus (OLP) is a chronic mucocutaneous inflammatory disorder that affects up to 4% of the worldwide population.^1^ In 2005, the World Health Organization classified OLP as a potentially malignant oral disorder,^2^ because 1.63% of lesions initially diagnosed as OLP evolved into oral squamous carcinomas.^3^ However, the etiology and pathogenesis of OLP are not well understood. Acanthosis is the frequent pathological change in OLP tissues.^4^, ^5^, ^6^ Besides the two features of OLP, a dense sub‐epithelium of mainly T lymphocytic infiltration and vacuolar degeneration of the basal layer, keratinocyte apoptosis is often found within or beneath the epidermis of OLP.^6^, ^7^, ^8^, ^9^ Although T cells are considered responsible for triggering apoptosis of basal epithelial cells via various pathways,^9^, ^10^, ^11^ vacuolar degeneration does not unequivocally indicate apoptosis.^12^ Moreover, down‐regulation of apoptosis markers (ie active caspase 3 and Bax)^4^, ^12^, ^13^ and up‐regulation of anti‐apoptotic markers (Bcl2)^14^ are frequently found in the basal cell layer of OLP. Thus, we proposed that abnormal apoptosis in the basal keratinocytes participates in the pathogenesis of OLP.

It is well‐known that apoptosis is mainly initiated by intrinsic or extrinsic pathways, which eventually regulate the activity of apoptotic proteins and/or anti‐apoptotic proteins, such as the proteins of the Bcl‐2 family.^15^, ^16^, ^17^ MicroRNAs (miRNAs) are small, non‐coding, single‐stranded RNAs that bind to complementary mRNA sequences and negatively regulate gene expression by repressing translation or destabilizing the target mRNAs.^16^ A single miRNA could broadly affect hundreds of target genes.^17^, ^18^, ^19^ Previous studies have reported that apoptosis is regulated by miRNAs in OLP through targeting apoptosis‐related proteins,^20^, ^21^ although the mechanism by which mRNAs are regulated in OLP is not clear.^3^

Increasing evidence has shown that miRNAs are involved in the regulation of OLP pathogenesis.^22^, ^23^ Ma et al^24^ reported that approximately 70 miRNAs were significantly up‐ or down‐regulated in OLP patients compared to healthy volunteers. Therefore, the current study aimed to clarify the role and mechanism of miRNAs in regulating apoptosis of OLP. Here, we showed that miR‐27b‐3p was significantly downregulated in oral biopsy specimens from OLP patients by using miRNA microarrays^25^ and RNA sequencing, and down‐regulation of miR‐27b‐3p reduced basal apoptosis in OLP. Moreover, we also found that cyclophilin D (CypD) was a new target of miR‐27b‐3p. In summary, we demonstrated a regulatory role of miR‐27b‐3p in OLP keratinocyte apoptosis.

## 2 MATERIALS AND METHODS

### 2.1 Cell culture

Human oral keratinocytes (HOKs) were cultured in RPMI‐1640 Medium (Invitrogen, Grand Island, NY) supplemented with 15% fetal bovine serum (FBS; PAA, Bentley, Western Australia), and HEK 293T cells were cultured in Dulbecco's Modified Eagle's Medium (Invitrogen) supplemented with 10% FBS and 1% penicillin‐streptomycin (Gibco, Rockville, MD). The methods for primary OLP keratinocyte culture have been described by Cao et al.^26^

### 2.2 Sample collection

Biopsy samples were collected from 25 cases of patients diagnosed with typically reticular OLP according to the 2003 World Health Organization diagnostic criteria^27^ at the Department of Oral Mucosal Diseases at Ninth People's Hospital, Shanghai Jiao Tong University School of Medicine (Shanghai, China). Clinical features on the collection of human biopsy samples including age, sex, area, size, and pattern of the lesions were extracted from the patient's medical files, and a control group of healthy control cases (25 cases) was selected. A biopsy sample of buccal mucosa approximately diameter 8 mm within the OLP lesion was obtained under sterile conditions and was divided into two parts for pathological examination and RNA sequencing (or cell culture). Normal mucosal tissues with no clinically visible inflammation were collected from retained wisdom teeth extraction operations and were used as healthy control samples. Paraffin‐embedded samples clinically and histologically diagnosed as reticular OLP were obtained from the Oral Pathology Department of Ninth People's Hospital, Shanghai Jiao Tong University School of Medicine, during a 4‐year period (2010‐2014). Clinical features of human biopsy samples for the study are shown in Table 1. Signed informed consent forms were acquired before biopsy and pathological examination. All experimental procedures were approved by the Research Ethics Committee of Shanghai Ninth People's Hospital (Approval number NSFC81400512).

**Table 1:** Clinical features of human biopsy samples for the study

Group	n	Age (mean ± SD)	Sex	Area	Size diameter (mm)	Pattern
			Male, n (%)			
		Range	Female, n (%)			
HC	25	34.28 ± 7.22	11 (44)	Buccal mucosa	8	－
		23‐48	14 (56)			
Oral lichen planus	25	43.72 ± 10.88	10 (40)	Buccal mucosa	8	Reticular
		28‐66	15 (60)			

### 2.3 TUNEL in situ detection of DNA fragmentation

In situ cell death detection kit (Roche, Berlin, Germany) (TUNEL technology) was used to analyse apoptosis in OLP and control samples. After deparaffinizing and dehydration, sections were incubated with proteinase K and 30% H_2_O_2_, respectively, followed by incubation with complete labelling reaction buffer and antibody solution at 37°C for 1 hour. The antigen‐antibody interaction was visualized by fluorescence labelling. Sections were then counter stained with DAPI, and visualized under a fluorescence microscope. At least five fields in each slide were analysed for TUNEL‐positive cells in each group. The evaluation was performed by an investigator blinded to the groups studied.

### 2.4 In situ hybridization analyses

After deparaffinizing and dehydration, each section was washed and subsequently treated with 15 µg/mL of proteinase K for 10 minutes at 37°C. The sections were then treated with 15× saline sodium citrate (SSC) for 15 minutes, and hybridized with a Digoxigenin (DIG)‐labelled oligonucleotide probe (Exqion, Vedbaek, Denmark) overnight at 37°C in buffer (50% formamide, 5× SSC, 5× Denhardt's solution, and 250 µg/mL of Baker's yeast transfer RNA). Afterward, sections were washed at 37°C in 2× SSC for 15 minutes, and 1× SSC for 30 minutes. Blocking was performed for 3 hours at 37°C with 2% goat serum and 2 mg/mL bovine serum albumin in phosphate‐buffered saline (PBS) and alkaline phosphatase‐conjugated Fab anti‐DIG antibody (Roche, Mannheim, Germany). Staining was performed using NBT/BCIP (Roche), with Nuclear Fast Red used as the counterstain. The level of miR‐27b‐3p expression was measured using Image Pro‐Plus software, version 6.0 (Media Cybernetics, Rockville, CA).^28^

### 2.5 Lentiviral packaging and the establishment of stable cell lines

To construct the miR‐27b‐3p overexpression (OE) and knockdown (KD) vectors, two oligonucleotides were respectively synthesized (Sangon Biotech, Shanghai, China). The primers were as follows: the forward primer for miR‐27b‐3p overexpression, 5′‐GATCCTTCACAGTGGCTAAGTTCTGCCTTCCTGTCAGAGGCAGAAGTTAGCCACTGTCAATTTTTG‐3′; the reverse primer, 5′‐AATTCAAAAATTGACAGTGGCTAACTTCTGCCTCTGACAGGAAGGCAGAACTTAGCCACTGTGAAG‐3′; the forward primer for miR‐27b‐3p knockdown, 5′‐GATCCGTTCACACTGGCTAAGTTCGGCCTTCCTGTCAGAGCAGAACTTAGCCACTGTGAATTTTTG‐3′; and the reverse primer, 5ʹ‐AATTCAAAAATTCACAGTGGCTAAGTTCTGCTCTGACAGGAAGGCCGAACTTAGCCAGTGTGAACG‐3ʹ). They were cloned into the EcoRI and BamHI sites in the miRZip vector (Open Biosystem, Pittsburgh, PA) after annealing.

The full‐length CypD cDNA fragment was cloned into the pLentiSin‐puro vector with the following primers: the sense primer for CypD OE was 5ʹ‐TTTGAATTCATGGATTACAAGGATGACGACGATAAGCTGGCGCTGCGCTGCGGCTC‐3ʹ; and the antisense primer was 5ʹ‐TTTGGATCCTTAGCTCAACTGGCCACAGT‐3ʹ. Three short‐hairpin RNA (shRNA) fragments specifically targeting human CypD (NM_005729.3) used the following primers: the sense primer for CypD KD1 was GATCCGACATCCAAGAAGATTGTCATCTTCCTGTCAGAATGACAATCTTCTTGGATGTCTTTTTG, and the antisense primer was AATTCAAAAAGACATCCAAGAAGATTGTCATTCTGACAGGAAGATGACAATCTTCTTGGATGTCG; the sense primer for CypD KD2 was GATCCCATGGCTAATGCTGGTCCTAACTTCCTGTCAGATTAGGACCAGCATTAGCCATGTTTTTG, and the antisense primer was AATTCAAAAACATGGCTAATGCTGGTCCTAATCTGACAGGAAGTTAGGACCAGCATTAGCCATGG; the sense primer for CypD KD3 was GATCCCGGCTACAAAGGCTCCACCTTCTTCCTGTCAGAAAGGTGGAGCCTTTGTAGCCGTTTTTG, and the antisense primer was AATTCAAAAACGGCTACAAAGGCTCCACCTTTCTGACAGGAAGAAGGTGGAGCCTTTGTAGCCGG. Primers were synthesized (Sangon Biotech) and inserted into the MirZip lentiviral shRNAmir vector. Overexpression lentiviruses were generated in 293T cells by the co‐transfection of a vector containing the gene or gene fragment in question, and using the psPAX2 and pMD2G plasmids. Knockdown lentiviruses were generated with a four plasmid packaging system, including pMirzip‐miR27b, pVSVg, pRSV‐Rev, and Pmdl(g/p), by incubating the cells for 72 hours. Viral stocks collected from the culture media of the transfected 293T cells were used to infect the HOK cells. The miR‐27b‐3p OE and KD, CypD OE, and KD in the HOK cell line were generated and compared with empty vector expressing cell lines.

### 2.6 Plasmid construction and dual luciferase reporter assay

The full‐length 3′‐UTR of the human CypD gene was amplified by PCR using human genomic DNA as a template. The sense primer was 5′‐TTTGAGCTCTCCTCACGACCTCATTTCTGGG‐3′, and the antisense primer was 5′‐TTTCTCGAGGCTCAGTAAAGATCAGCTCCAA‐3′. Site‐directed mutagenesis was performed to generate a mutant CypD 3′‐UTR that contained mutations in the conserved miR‐27b‐3p binding site. In the mutant 3′‐UTR of the CypD gene, the nucleotide sequence complementary to nucleotides 2‐5 of the miR‐27b‐3p binding site (ACUGUGA) was mutated to the sequence found in miR‐27b‐3p (TCAGAGT). The HEK 293T cells were seeded in 12‐well plates at a density of 2 × 10^5^ cells/well and co‐transfected with 200 ng of pmirGLO‐*cypd* 3ʹ‐UTR (wild‐type or mutant) plasmid and 3000 ng of Scr‐miR or miR‐27b‐3p mimics using the Lipofectamine 2000 reagent (Invitrogen) according to the manufacturer's protocol. After 48 hours of incubation, the luciferase activity was measured using the dual‐luciferase reporter assay system (Promega, Beijing, China).

### 2.7 Oligonucleotide transfection

The miR‐27b‐3p mimics (double strand RNA) and inhibitor (single strand RNA) were synthesized by GenePharma (Shanghai, China). Synthetic mimics or inhibitors were transfected into cell cultures using Lipofectamine 2000 (Invitrogen) to promote or inhibit miR‐27b‐3p activity, respectively. Negative controls were used to validate both reactions. The final concentration of the mimics and inhibitors was 100 nmol/L and 200 nmol/L, respectively.

### 2.8 Cell viability assays

To test the optimal concentration of etoposide for the induction of apoptosis in HOK cells, cell viability was analysed by the median effect equation to obtain the IC_50_ value based on all the data points of the cytotoxicity‐concentration curve. Cells (5000 cells/well) were seeded into a 96‐well plate and treated with etoposide at different concentrations (10×, 1×, 1:10, 1:100, 1:1,000, and 1:10,000); the cells were labelled with CCK8 reagent (Dojindo Molecular Technologies, Tokyo, Japan) for 2 hours. Cell viability was measured at an absorbance at 450 nm using a microplate reader (Model 450; Bio‐Rad Laboratories, Hercules, CA). Based on the survival ratio of cells, the IC_50_ value was obtained by the integration of data from the cells treated with the etoposide gradient, thereby generating quantitative measures of protection.

### 2.9 RNA isolation and qPCR

Total RNA was extracted from the cultured cells using TRIzol^®^ reagent (Invitrogen) according to the manufacturer's instructions. The reverse transcription of miR‐27b‐3p was performed using the TaqMan^®^ MicroRNA RT kit (Invitrogen). The primer sequence used for the cDNA synthesis was GTCGTATCCAGTGCAGGGTCCGAGGTATTCGCACTGGATACGACGCAGAA. For the reverse transcription of CypD mRNA, 2 μg of total RNA from each sample was transcribed into cDNA using the PrimerScript RT Reagent Kit (Takara, Dalian, China). For qPCR analysis of miR‐27b‐3p, the forward primer sequence was 5′‐CGGCGGTTCACAGTGGCTAA‐3′, and the reverse primer was 5′‐GTGCAGGGTCCGAGGT‐3′. For qPCR analysis of CypD, the forward primer was 5′‐AGACAGACTGGTTGGATGGC‐3′ and the reverse primer was 5′‐TGGGCACACGTATCGTTTCA‐3′. The reaction mixtures were incubated at 16°C for 30 minutes, 42°C for 30 minutes, 85°C for 5 minutes, and then held at 4°C overnight. The qPCR was performed on a real‐time PCR system (ABI 7500 Fast; Applied Biosystems, Carlsbad, CA) according to the manufacturer's protocol. The RNU48 mRNAs and β‐actin were used as the internal controls. The forward primer for RNU48 was 5′‐TGATGATGACCCCAGGTAACTC‐3′, and the reverse was 5′‐GAGCGCTGCGGTGATG‐3′. The forward primer for β‐actin was 5′‐GCGGGAAATCGTGCGTGACATT‐3′ and the reverse was 5′‐GATGGAGTTGAAGGTAGTTTCG‐3′.

### 2.10 Protein extraction and Western blot analysis

To investigate the optimum time for etoposide‐induced apoptosis, cells were treated with etoposide for 0, 6, 12, 24, 36, and 48 hours. Total protein extract was prepared using RIPA lysis buffer containing 1× protease inhibitor mixture (Roche, Basel, CH, Switzerland) and 1× PMSF. Equivalent amounts of protein (20‐40 μg) from each sample were resolved on 8%–12% SDS‐polyacrylamide gels and transferred by electroblotting to PVDF membranes (Bio‐Rad). The membranes were blocked with 5% non‐fat dry milk in TBST for 1 hour and incubated overnight at 4°C in 5% BSA in TBS with antibodies against CypD (Santa Cruz Biotechnology, Santa Cruz, CA), Bcl2, Apoptosis Antibody Sampler Kit (for PARP, cleaved caspase 3, cleaved caspase 9, and caspase 9) (Cell Signaling Biotechnology, Beverly, MA). The antibody against β‐actin (Santa Cruz Biotechnology) was used for the internal control.

### 2.11 Apoptosis assay

Apoptosis was assessed using annexin‐V‐propidium iodide (PI). At the end of the incubation period, floating as well as adherent cells were harvested by trypsinization and washed with PBS. Cells were stained with 5 µL of annexin V in 60 µL 1× binding buffer for 15 minutes at room temperature in the dark. After staining, 120 µL of 1× binding buffer and 5 µL of PI were added to the cell suspension and the cells were analysed using a FACScalibur flow cytometer (BD, Franklin Lakes, NJ).

### 2.12 Isolation of mitochondria

Mitochondria were isolated from cells as described in the Cell Mitochondria Isolation Kit instructions (Beyotime Biotech, Peking, China). Briefly, cells were harvested and centrifuged at 600 g at 4°C for 5 minutes. The pellets were dissolved in 20 mmol/L N‐2‐hydroxyethylpiperazine‐N0‐20‐ethanesulfonic acid (HEPES) buffer containing a protease inhibitor cocktail, and disrupted with a glass tissue grinder. Homogenates were centrifuged at 600 g at 4°C for 5 minutes, and the resulting supernatants were transferred to 0.5 mL conical tubes, and further centrifuged at 11 000 g at 4°C for 10 minutes, the resulting precipitates were the isolated mitochondria.

### 2.13 Co‐immunoprecipitation

Samples were homogenized in ice‐cold ‘immunoprecipitation buffer’ containing 20 mmol/L Tris‐HCl, pH 7.5, 150 mmol/L NaCl, and 1.5% Nonidet P‐40 supplemented with protease inhibitors. The extracts (500 µg protein per sample) were pre‐cleared with protein A/G beads and then mixed with non‐specific IgG (1 µg) or polyclonal mouse anti‐Bcl2 antibody (Cell Signaling Biotechnology), or anti‐CypD (Santa Cruz Biotechnology) overnight at 4°C, followed by the addition of 40 µL of protein A/G‐agarose (Santa Cruz Biotechnology) for 3 hours at 4°C. Immune complexes were washed four times in an ‘immunoprecipitation wash buffer’ (100 mmol/L Tris‐HCl, pH 7.5, 100 mmol/L NaCl, 0.1% Triton X‐100) and resuspended in 2× Laemmli buffer (Santa Cruz Biotechnology). The inputs were also mixed with 2× Laemmli buffer, and then the immunoprecipitation reactions and inputs were boiled for 10 minutes and pelleted in a centrifuge. The supernatants were subjected to Western blot analysis as described above.

### 2.14 Enzyme‐linked immunosorbent assay

Quantitation of CytC release was performed using an enzyme immunoassay technique and an Immunoassay Kit (eBioscience, San Diego, CA) according to the manufacturer's instructions. HOK cells were incubated with etoposide at 154.79 µg/mL for 24 hours, and the samples were pelleted, and supernatants were collected to measure the concentration of CytC.

### 2.15 Immunohistochemistry

Paraffin‐embedded clinical samples were deparaffinized, rehydrated, and blocked for endogenous peroxidase activity with 3% H_2_O_2_/methanol. After rinsing, tissue sections were blocked for non‐specific binding with 1% goat serum in PBS. Tissue slides were incubated with primary antibodies at 4°C overnight, followed by incubation with peroxidase‐labelled anti‐mouse secondary antibodies. Finally, antibody binding was visualized with a DAB substrate (ImmunoPure^®^; Pierce Biotechnology, Waltham. MA).

### 2.16 Statistical analysis

The data are presented as the mean ± SEM of at least three independent experiments. The samples were analysed using a two‐tailed unpaired Student's *t* test, unless otherwise noted, and multiple group comparisons were made using one‐way analyses of variance (ANOVA). *P* < 0.05 were considered statistically significant. *P* < 0.05, <0.01, and < 0.001 are indicated with one, two, and three asterisks (*, **, or ***), respectively. Graph Pad Prism software (San Diego, CA, USA; version 6.03) was used for data analyses.

## 3 RESULTS

### 3.1 MiR‐27b‐3p was significantly downregulated in the epithelial of OLP patients

**Figure 1:**
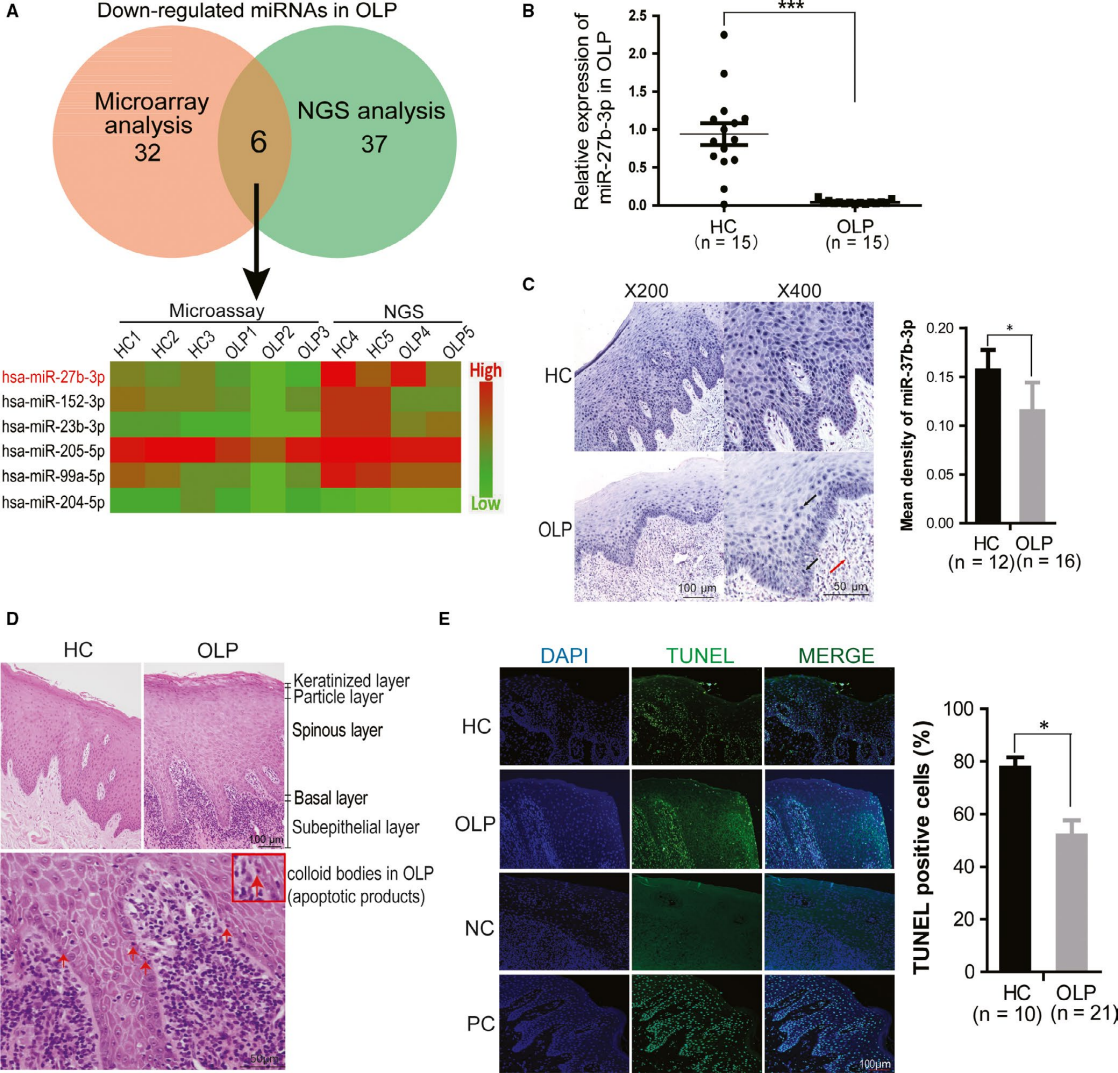
MiR‐27b‐3p was significantly down‐regulated in basal epithelia of oral lichen planus (OLP) tissues. (A) The miRNAs were screened by miRNA microarrays and RNA sequencing; the most down‐regulated miRNAs in OLP were presented, and healthy volunteer tissues were considered as controls. (B) MiR‐27b‐3p was verified by qPCR analyses. (C) MiR‐27b‐3p expression was detected by in situ hybridization. The blue represents the staining of miR‐27b‐3p; the red indicates nuclear staining. Left, magnification is ×200; Right, magnification is ×400. (D) Haematoxylin and eosin staining of OLP and healthy tissues, with colloid bodies visible in the OLP basal layer. Upper, magnification is ×200; below, magnification is ×400. (E) The TUNEL‐positive cells were analysed in the OLP and healthy control tissues, and the percentages of TUNEL‐positive cells in both groups were quantified. Magnification is ×200. HC, healthy control; NC, negative control; PC, positive control. *P < 0.05, ***P < 0.001

To determine what miRNAs are involved in OLP, the miRNA profiling was first performed by using miRNA microarrays (3HC vs 3OLP) in oral biopsy specimens of OLP patients. As shown in Figure 1, 38 miRNAs were found to be down‐regulated more than 2‐fold in OLP specimens, compared to that of healthy control specimens. Furthermore, the miRNA expression was examined in OLP specimens by using RNA sequencing. Forty‐three miRNAs were found to be down‐regulated more than 2‐fold in OLP specimens as shown by RNA sequencing (2HC vs 2OLP) (Figure 1). By comparatively analysing two datasets from microarrays and RNA sequencing, the miR‐27b‐3p was the most significantly down‐regulated miRNA (more than 3.6‐fold) among six commonly down‐regulated miRNAs (Figure 1 and Figure S1A). The miR‐27b‐3p expression was further verified in oral biopsies of OLP patients by using quantitative PCR (qPCR) and in situ hybridization (ISH) (Figure 1,C). Both methods showed that the miR‐27b‐3p levels decreased in OLP tissues compared to that of healthy control tissues. Moreover, the ISH data clearly revealed that the miR‐27b‐3p was mainly expressed in the epithelial layer, and was down‐regulated in OLP tissues, compared to healthy tissues (Figure 1).

To investigate the overall apoptosis levels in OLP tissues, haematoxylin and eosin staining (H＆E) and TUNEL (TdT‐mediated dUTP nick end labelling) assays were performed. As shown in Figure 1, the oral mucosal epithelium contained four layers from the surface: a keratinized layer (the most superficial layer), a particle layer (2‐3 layers of flat cells), a spinous layer (mainly comprised of epithelium), and a basal layer (cubic or dwarf columnar cells). The colloid bodies and the acanthosis were also seen in OLP tissues (Figure 1). The colloid bodies represent apoptotic keratinocytes. Using TUNEL analyses, both OLP and healthy tissues showed apoptotic cells with typically condensed nuclei, and the number of apoptotic cells decreased in the epithelial layer in OLP tissues compared to healthy tissues (Figure 1 and Figure S1B). Moreover, the apoptotic cells were mainly located in the basal layer of OLP specimens, suggesting that apoptosis was suppressed in the basal layer.

### 3.2 Down‐regulation of miR‐27b‐3p suppressed keratinocytes apoptosis

**Figure 2:**
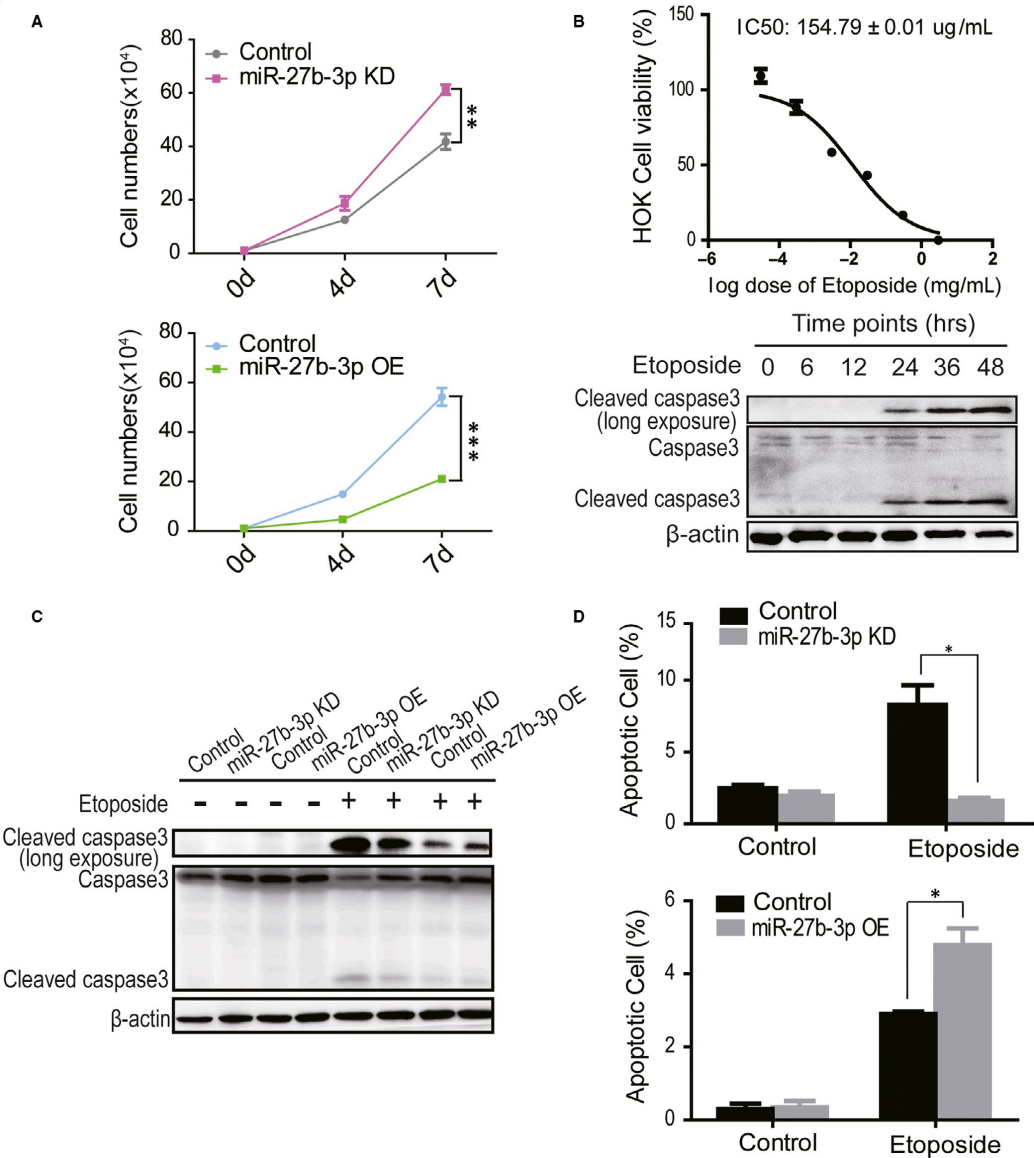
Down‐regulation of miR‐27b‐3p suppressed epithelium apoptosis. (A) The effects of miR‐27b‐3p knockdown or overexpression on HOK cell growth analysed by cell counting assays. (B) The IC_50_ of etoposide for HOK cells was determined by CCK8 assays, and the cleaved caspase levels for etoposide‐induced apoptosis on HOK cells were determined by Western blotting. (C) The effects of miR‐27b‐3p on the cleavage of caspase3, determined by Western blots in HOK cells. (D) The effect of miR‐27b‐3p on annexin V turnover was analysed by flow cytometry in HOK cells. The results are representative of three independent experiments and are presented as the mean ± SEM. *P < 0.05, **P < 0.01, ***P < 0.001

To investigate the role of miR‐27b‐3p in OLP tissues, miR‐27b‐3p was either overexpressed or knocked down in human oral keratinocytes (HOKs), mediated by lentiviruses infection. The HOK cells are immortalized epithelial cells from the basal layer of oral mucosa tissues.^29^ The survival ability of keratinocytes was first analysed in empty vector‐transfected control cells and miR‐27b‐3p overexpressing/knockdown cells. As shown in Figure 2, knockdown of miR‐27b‐3p promoted cell growth; in contrast, overexpression of miR‐27b‐3p inhibited cell growth.

To further investigate the regulation of miR‐27b‐3p on apoptosis, apoptotic signalling was determined in the HOK cells overexpressing or depleted of miR‐27b‐3p. The cleaved caspase 3 levels were determined in the HOK cells treated with etoposide, a compound known to trigger the caspase 3‐mediated apoptotic pathway.^30^, ^31^ As shown in Figure 2, etoposide treatment induced caspase 3 cleavages. The expression of apoptotic markers was analysed in HOK cells with overexpressing or depleted of miR‐27b‐3p by Western blot, knockdown of miR‐27b‐3p reduced both cleaved caspase 3 and cleaved PARP (poly ADP‐ribose polymerase) and overexpression of miR‐27b‐3p promoted the cleavage (Figure 2 and Figure S1C). The percentage of cells undergoing apoptosis was also analysed in HOK cells with overexpressing or depleted of miR‐27b‐3p by flow cytometry, indicating knockdown of miR‐27b‐3p reduced annexin V turnover ratio, from 12.84% to 2.05%. In contrast, overexpression of miR‐27b‐3p increased the percentage of annexin V positive cells, from 3.60% to 17.10% (Figure 2), suggesting that miR‐27b‐3p promoted keratinocytes apoptosis and down‐regulation of miR‐27b‐3p probably contributed to the acanthosis of the epithelial layer.

### 3.3 MiR‐27b‐3p regulated CypD expression

**Figure 3:**
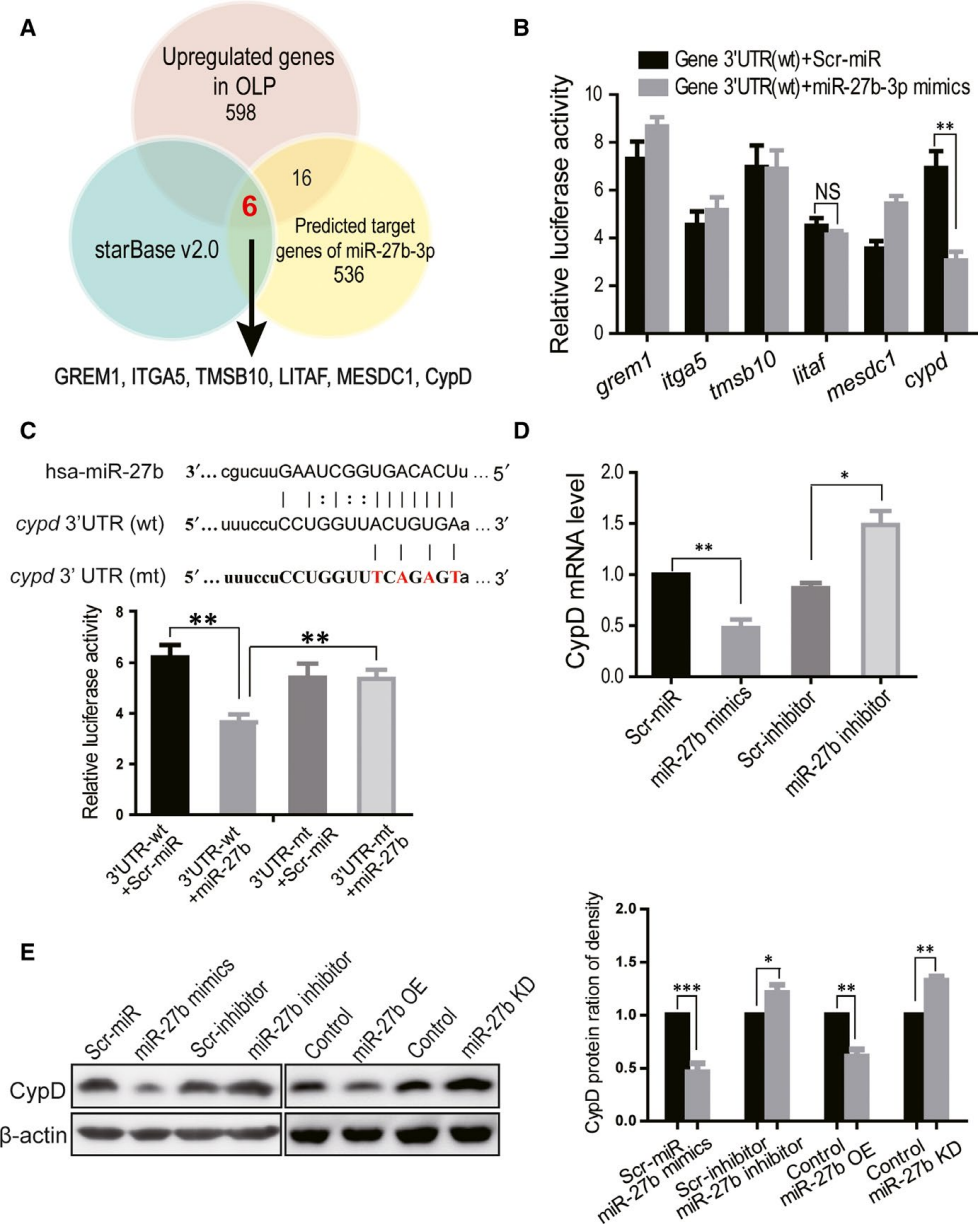
The miR‐27b‐3p regulates CypD expression. (A) Screening of miR‐27b‐3p target genes by integrative analyses using the web‐based prediction and RNA sequencing data. (B) The verification of six candidate genes using the 3′‐UTR luciferase assay. Plasmids containing wild‐type 3′‐UTRs of all six genes were separately co‐transfected with the miR‐27b‐3p mimics or scrambled miRNA. (C) The effects of the miR‐27b‐3p on wild‐type UTR or mutant UTR were determined by the 3′‐UTR luciferase assay. The key complementary sequence of CypD 3′‐UTR to miR‐27b‐3p was highlighted, and the nucleotides in red were mutated to their complementary nucleotides. (D) The effects of miR‐27b‐3p mimics or inhibitors on CypD expression in HOK cells were detected by q‐PCR and (E) Western blot analyses, respectively, and were quantitated (right). Results are representative of three independent experiments and are presented as the mean ± SEM. *P < 0.05, **P < 0.01, ***P < 0.001

To determine the mechanism by which miR‐27b‐3p promoted keratinocyte apoptosis, a web‐based prediction program (http://starbase.sysu.edu.cn) was used to identify downstream targets of miR‐27b‐3p. In total, 536 potential target genes of miR‐27b‐3p were selected by Targetscan software, including RNA22, PicTar5, PITA, and miRanda.^32^ After integrative analyses with up‐regulated genes in OLP samples screened by RNA sequencing, six genes including *cypd*, *grem1*, *litaf*, *tmsb10*, *itga5*, and *mesdc1* were overlapping in two datasets and were selected for further verification (Figure 3).

To further determine what genes were targets of miR‐27b‐3p, the 3′‐UTR luciferase assay was performed. As shown in Figure 3, miR‐27b‐3p overexpression only reduced the luciferase activity of the reporter gene with *cypd* 3′‐UTR, but not the 3′‐UTRs of the other five genes. When the miR‐27b‐3p binding sequence ACUGUGA was mutated into TCAGAGT on the *cypd* 3′‐UTR, overexpression of miR‐27b‐3p no longer suppressed luciferase activity (Figure 3). Moreover, the expression of CypD was analysed by qPCR and Western blotting in HOK cells transfecting miR‐27b‐3p mimics or miR‐27b complementary oligonucleotides (inhibitor). Both qPCR and Western blotting results showed that miR‐27b‐3p mimics decreased CypD expression in HOK cells. In contrast, miR‐27b‐3p inhibitors increased CypD expression (Figure 3,E). When miR‐27b‐3p was stably overexpressed or knocked down by lentiviruses, CypD protein levels were also decreased or increased in HOK cells, respectively (Figure 3). Taken together, these results showed that miR‐27b‐3p negatively regulated CypD expression in keratinocytes cells.

### 3.4 CypD suppressed apoptosis by increasing Bcl2 stability

**Figure 4:**
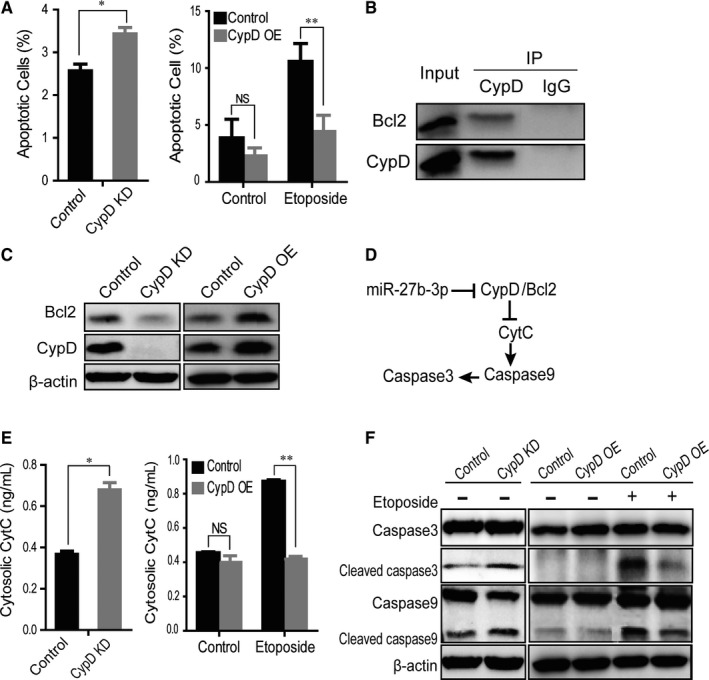
CypD suppressed apoptosis by increasing Bcl2 stability. (A) The effect of CypD on apoptosis of epithelial cells with or without etoposide was determined by the annexin V turnover analysis. (B) The Bcl2 was co‐immunoprecipitated with CypD. Mitochondrial proteins were extracted from HOK cells. (C) Bcl2 protein was detected in epithelial cells overexpressing or depleted of CypD. (D) The possible signalling pathway involved in this study. (E) The effect of CypD on CytC release. CytC was examined by enzyme‐linked immunosorbent assay assays in HOK cells depleted of CypD or treated with etoposide. The etoposide concentration was 154.79 ± 0.01 μg/mL. (F) The effects of CypD on caspase 3/9 cleavage. HOK cells overexpressing or depleted of CypD were treated for 24 h with etoposide at a concentration of 154.79 ± 0.01 μg/mL. The results are representative of three independent experiments and are presented as the mean ± SEM. **P < 0.01, ***P < 0.001

To determine the effects of CypD on apoptosis in keratinocytes, annexin V turnover ratios were analysed by flow cytometry in HOK cells expressing or depleted of CypD. As shown in Figure 4, knockdown of CypD increased the percentage of annexin V‐positive cells (*P* < 0.05). In contrast, overexpression of CypD reduced the percentage of annexin V‐positive cells, especially when the cells were treated with etoposide, making this change more obvious (*P* < 0.01) (Figure 4).

To determine the mechanism by which CypD regulated keratinocyte apoptosis, co‐immunoprecipitation was performed using CypD antibody to determine whether CypD regulated apoptosis by interacting with Bcl2 (a major anti‐apoptotic protein).^31^ As shown in Figure 4, Bcl2 co‐immunoprecipitated with CypD in a mitochondrial extract of HOK cells. Moreover, we found that the protein levels of Bcl2 increased in CypD overexpressing HOK cells, and vice versa (Figure 4), suggesting that CypD inhibited apoptosis by regulating Bcl2 protein stability.

Because Bcl2 is known to regulate apoptosis through cytochrome C (CytC) release and caspase 3/9 activation (Figure 4), cytosolic CytC concentration, and cleaved caspase 3/9 levels were analysed to further determine whether CypD regulated apoptosis through Bcl2. The enzyme‐linked immunosorbent assay (ELISA) results showed that the knockdown of CypD significantly increased the CytC release from mitochondria. In contrast, overexpression of CypD reduced the etoposide‐induced CytC release (Figure 4). Subsequently, Western blotting results showed that knockdown of CypD increased the level of cleaved caspase 3/9, and overexpression of CypD decreased caspase 3/9 cleavage, especially when cells were treated with etoposide (Figure 4), suggesting that Bcl2 mediated CypD regulation of apoptosis.

### 3.5 CypD was up‐regulated in OLP tissues and was associated with the prognosis of oral squamous carcinoma

**Figure 5:**
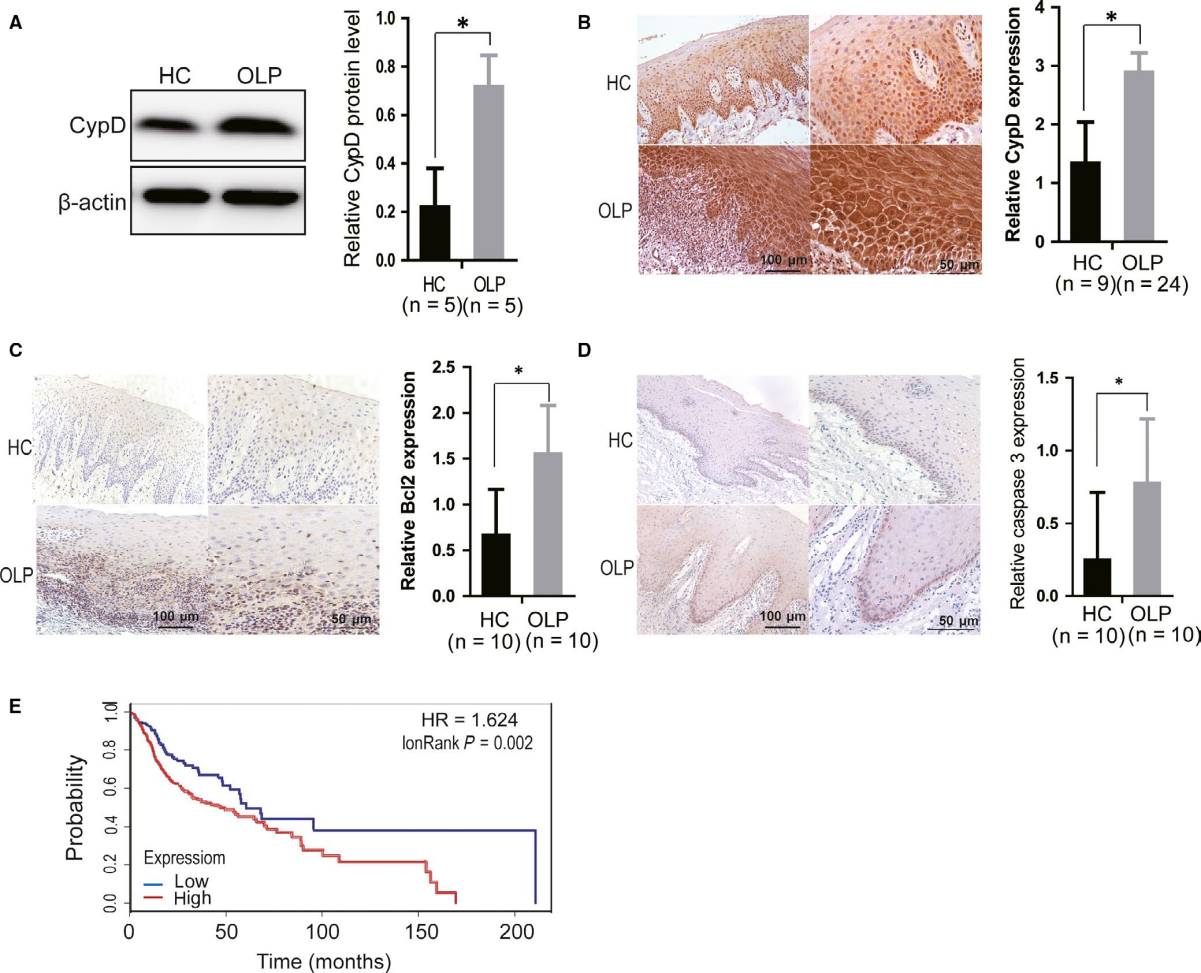
CypD was up‐regulated in oral lichen planus (OLP) and was a prognostics marker for oral squamous carcinoma. (A) The protein levels of CypD were detected in cultured primary epithelial cells from OLP and healthy control (HC) mucosa by Western blots and further analysed by densitometry. The data were normalized to β‐actin. (B, C and D) CypD, Bcl2, and caspase3 expression were analysed by immunohistochemical staining of OLP and healthy control (HC) tissues. The expressions were further analysed by densitometry. Left, magnification is ×200; Right, magnification is ×400. (E) The overall survival of oral squamous carcinoma patients was analysed. The patients were divided into two groups according to low expression (n = 149) and high expression (n = 370) of CypD. *P < 0.05

To determine whether CypD was a novel biomarker of OLP, the protein level of CypD was first analysed by Western blotting in primary keratinocytes from patients with OLP and from healthy controls. As shown in Figure 5, CypD was up‐regulated in primary keratinocytes from OLP patients, compared to that from healthy controls. The immunohistochemistry staining assay results also showed that protein levels of CypD were up‐regulated in OLP tissues (Figure 5), which was consistent with the data using miR‐27b‐3p. In addition, the immunohistochemistry staining assay showed increased Bcl2 and caspase 3 expression levels in OLP tissues and there was statistical significance (Figure 5,D), which further confirmed the corresponding results using miR‐27b‐3p in vitro, except that there was no statistical significance in caspase 9 expression level (Figure S1D), which may be due to the interference of various apoptotic regulatory pathways.

Because the OLP potentially develops into malignant oral squamous carcinoma at a late stage, finding a prognostic marker is critical for OLP patients. To further determine whether CypD is a prognostic marker for oral squamous carcinoma, survival analyses were performed on patients with high expression or low expression of CypD. As shown in Figure 5, patients with low expression of CypD survived for a longer period than those with high expression of CypD, suggesting that CypD was a prognostic marker for oral squamous carcinoma.

## 4 DISCUSSION

OLP is thought to undergo premalignant transformation,^33^, ^34^ however, the mechanisms underlying the pathogenesis of OLP still remain largely unknown. Recently, increasing evidence indicates the important roles of the miRNAs in the pathology of OLP. Our results demonstrated the significant down‐regulation of miR‐27b‐3p in tissues of OLP patients compared to those of control individuals, suggesting that miR‐27b‐3p may be involved in the pathogenesis of OLP. Similarly, Aghbari et al^35^ showed a significant decrease in miR‐27b in OLP, which was consistent with our results and the previous observations.^25^

We also observed colloid bodies and decreased apoptosis in OLP epithelium, and further found that apoptotic cells were mainly located in the basal layer of OLP. Abnormal apoptoses, such as in apoptotic keratinocytes and colloid bodies, are often found in OLP patients,^6^, ^8^, ^36^ specifically in the basal layer.^19^, ^37^ González‐Moles et al^38^ reported that apoptosis was not consistently present in all OLP tissues, as evidenced by the differential expression of caspase 3 and Bcl2, further suggesting that pathological changes might be heterogeneous in different layers of OLP tissues. Moreover, our data showed increased Bcl2 and caspase 3 expression levels in OLP, especially in the basal layer of OLP epithelium, indicating suppressed apoptosis in the basal layer, which may contribute to the acanthosis of OLP.

In our study, we found that CypD was a downstream target of miR‐27b‐3p that was up‐regulated in the epithelial layer of OLP. CypD, also known as peptidyl prolyl isomerase F, regulates the mitochondrial permeability transition pore opening,^39^ which leads to mitochondrial swelling, outer membrane rupturing, and the release of apoptotic mediators into the cytoplasm,^14^, ^40^ including cytochrome C. Although CypD might promote apoptosis by inducing mitochondrial permeability transition (MPT) pore openings,^39^, ^41^, ^42^ recent studies reported that CypD suppressed apoptosis in various tumour cells through distinct signalling pathways.^43^, ^44^, ^45^ Eliseev and co‐workers^31^ reported that CypD suppressed apoptosis by interacting with Bcl2, which was consistent with our current findings that up‐regulated CypD expression was accompanied with reduced apoptosis in the basal epithelial layer of OLP tissues. Overexpression of CypD promoted keratinocyte resistance to apoptosis by associating with Bcl2 in the OLP epithelium, with the latter inhibiting cytochrome C release and caspase 3/9 activation, which was supported by the results from Eliseev et al.^31^

Our results demonstrated that miR‐27b‐3p down‐regulation as well as CypD up‐regulation inhibited basal keratinocyte apoptosis, suggesting that miR‐27b‐3p/CypD‐medicated apoptosis may interfere with the pathogenesis of OLP. Jia et al^46^ demonstrated that histone deacetylase 6 regulated miR‐27b that suppressed proliferation, promoted apoptosis, and targeted oncogenic MET, implicating the potential application of miR‐27b for prognostic prediction and therapy of diffuse large B‐cell lymphoma. Moreover, miR‐27b‐3p also inhibited the inflammatory response by reducing the expression/secretion of inflammatory factors, such as IL‐1A, JAK2, IL‐6, and IL‐1B,^47^ suggesting that besides apoptosis, miR‐27b‐3p/CypD signalling may play multiple roles in oral mucosal tissue; however, the specific mechanism needs further study in the future.

In summary, our study showed that miR‐27b‐3p regulated keratinocyte apoptosis of the basal epithelial in OLP. MiR‐27b‐3p down‐regulation directly increased CypD protein levels; the latter inhibited keratinocyte apoptosis by binding to Bcl2. Further study of the miR‐27b‐3p/CypD signalling complex may lead to a better understanding of molecular pathways involved in the pathogenesis of OLP and epithelial apoptosis, thus facilitating the development of more effective therapies. However, more studies are necessary to thoroughly elucidate the role of miR‐27b‐3p in OLP.

## CONFLICT OF INTEREST

The authors have no conflict of interest to declare.
