# Supplementary figures and images for: Down‐regulation of miRNA‐27b‐3p suppresses keratinocytes apoptosis in oral lichen planus
Source: J Cell Mol Med. 2019 Apr 11;23(6):4326–37. doi: 10.1111/jcmm.14324 (PMC6533518; doi:10.1111/jcmm.14324)

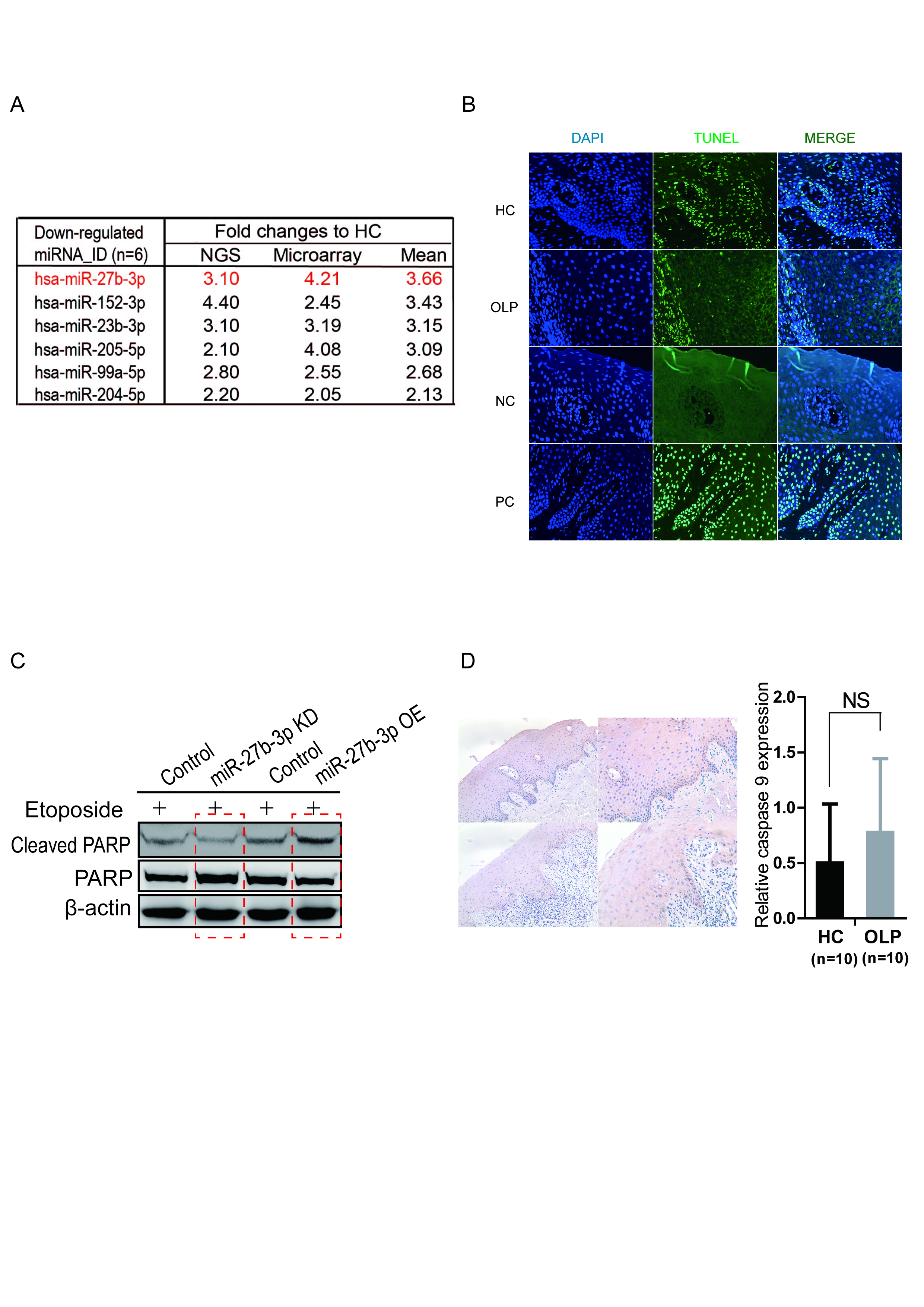

Supplement: Supplementary file 1 [file JCMM-23-4326-s001.tif]
